# Supplementary material for: The low survival rate of European hare leverets in arable farmland: evidence from the predation experiment
Source: PeerJ. 2024 Apr 30;12:e17235. doi: 10.7717/peerj.17235 (PMC11067912; doi:10.7717/peerj.17235)

**Supplementary material**

**S1 Fig.** Map of study area and location of individual study plots in Northern and Central Bohemia, Czech Republic. Image source credit: [Geoportal ČÚZK](https://geoportal.cuzk.cz/(S(r4rivavp0sorwqgxvpwswujp))/Default.aspx?lng=EN&mode=TextMeta&metadataID=CZ-CUZK-ORTOFOTO-R&metadataXSL=full&side=ortofoto), [CC BY 4.0](https://creativecommons.org/licenses/by/4.0/)
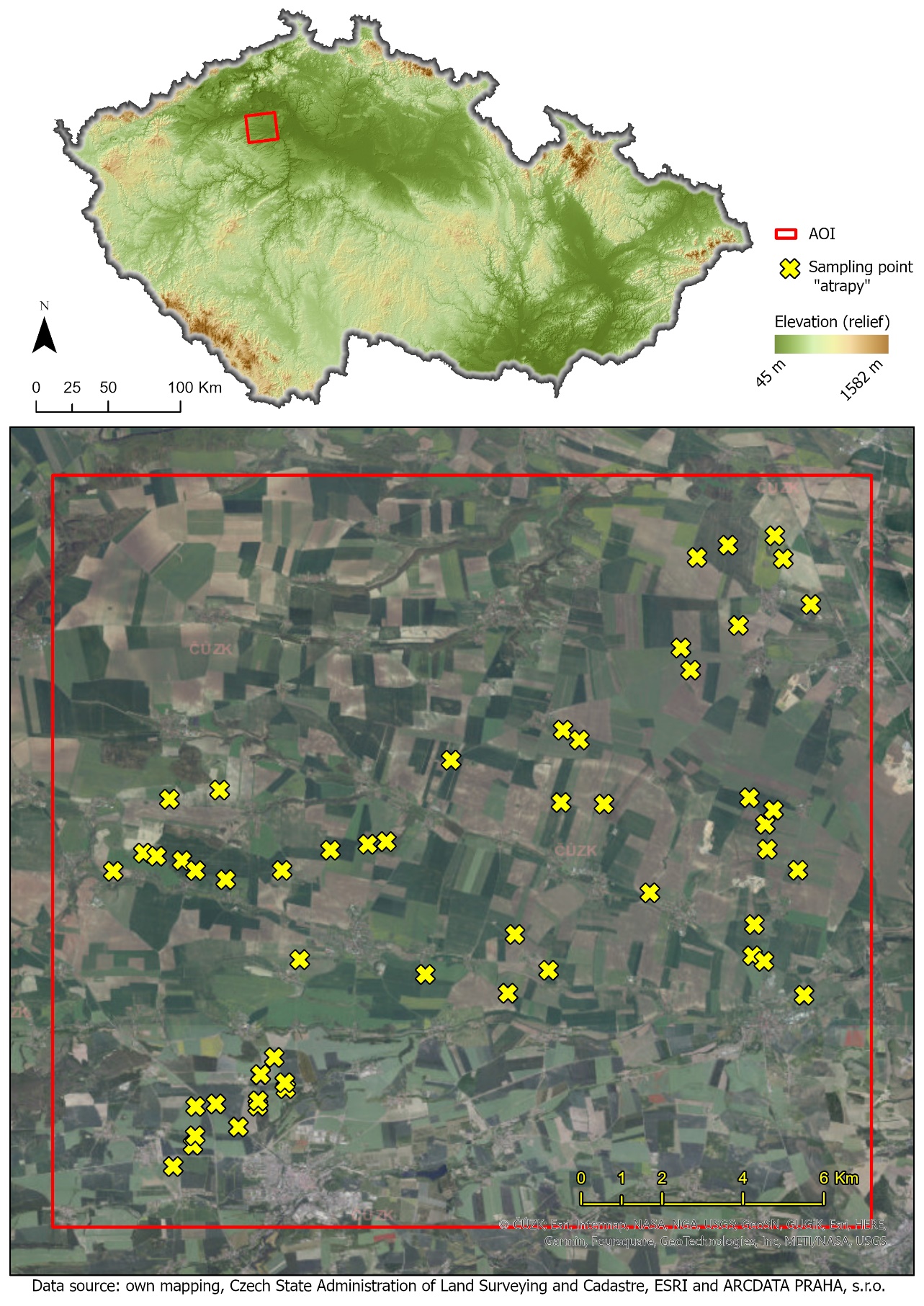


Note: Free orthophoto map used as a background of S1 Fig. is available at www.cuzk.cz/en.

**S2 Fig.** Dummy juvenile hare made from 15 × 5 cm hare skin in the winter crop field.


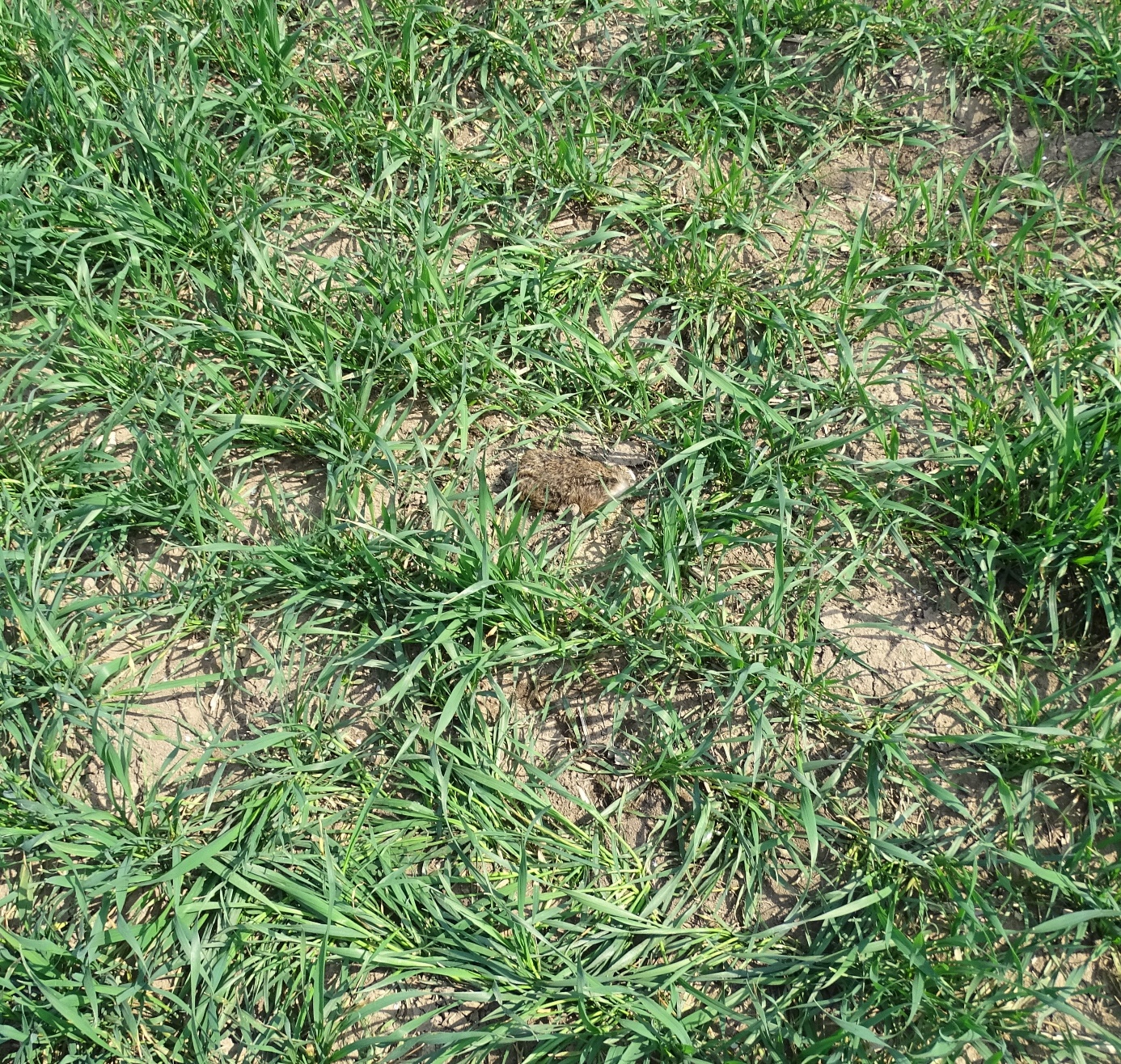


**S3 Fig.** Numbers of predation events based on predation order for each species (n = 44).


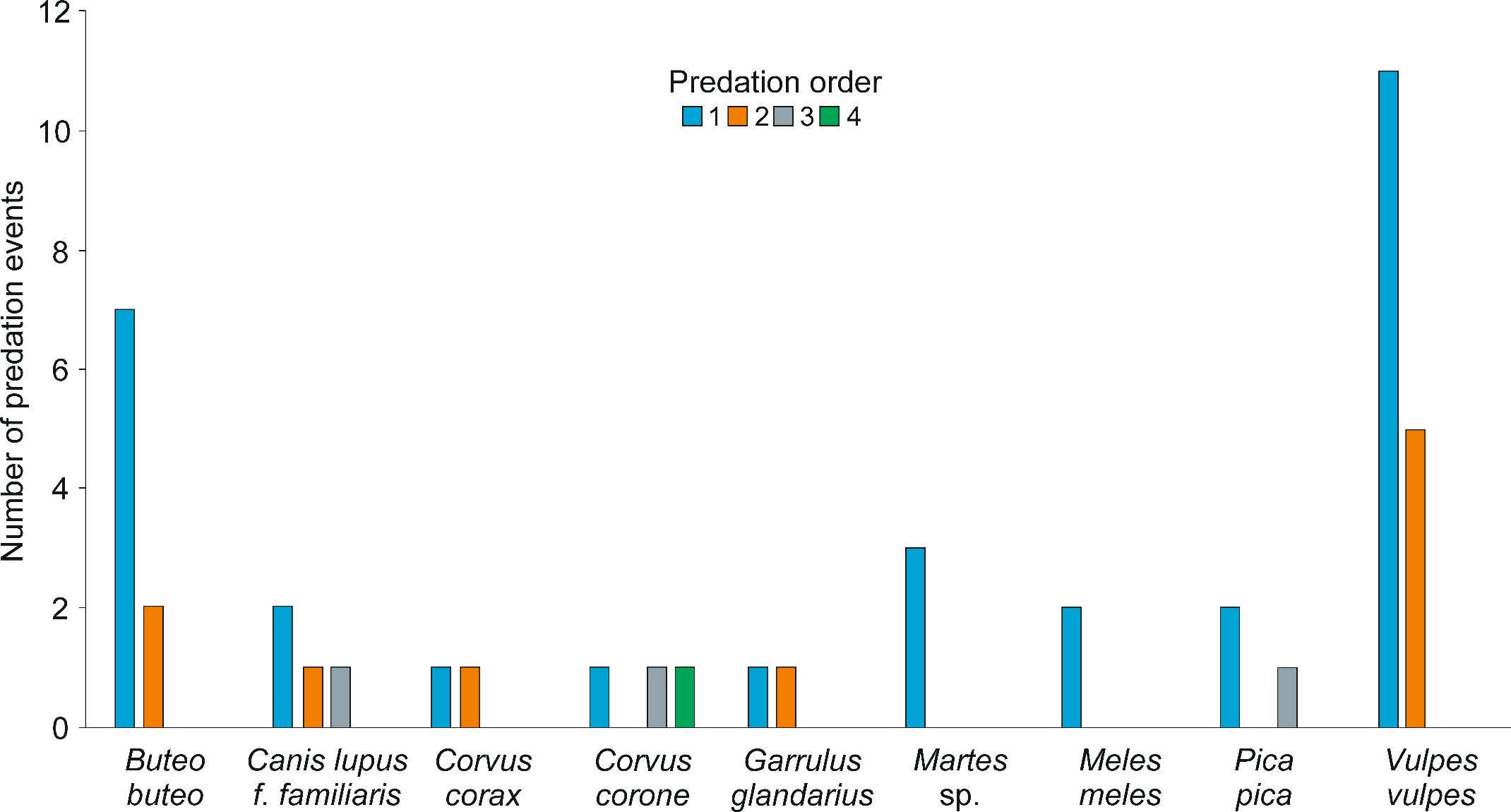


**S4 Fig.** Numbers of predation events during the day/night according to predator type (bird/mammal, n = 46).


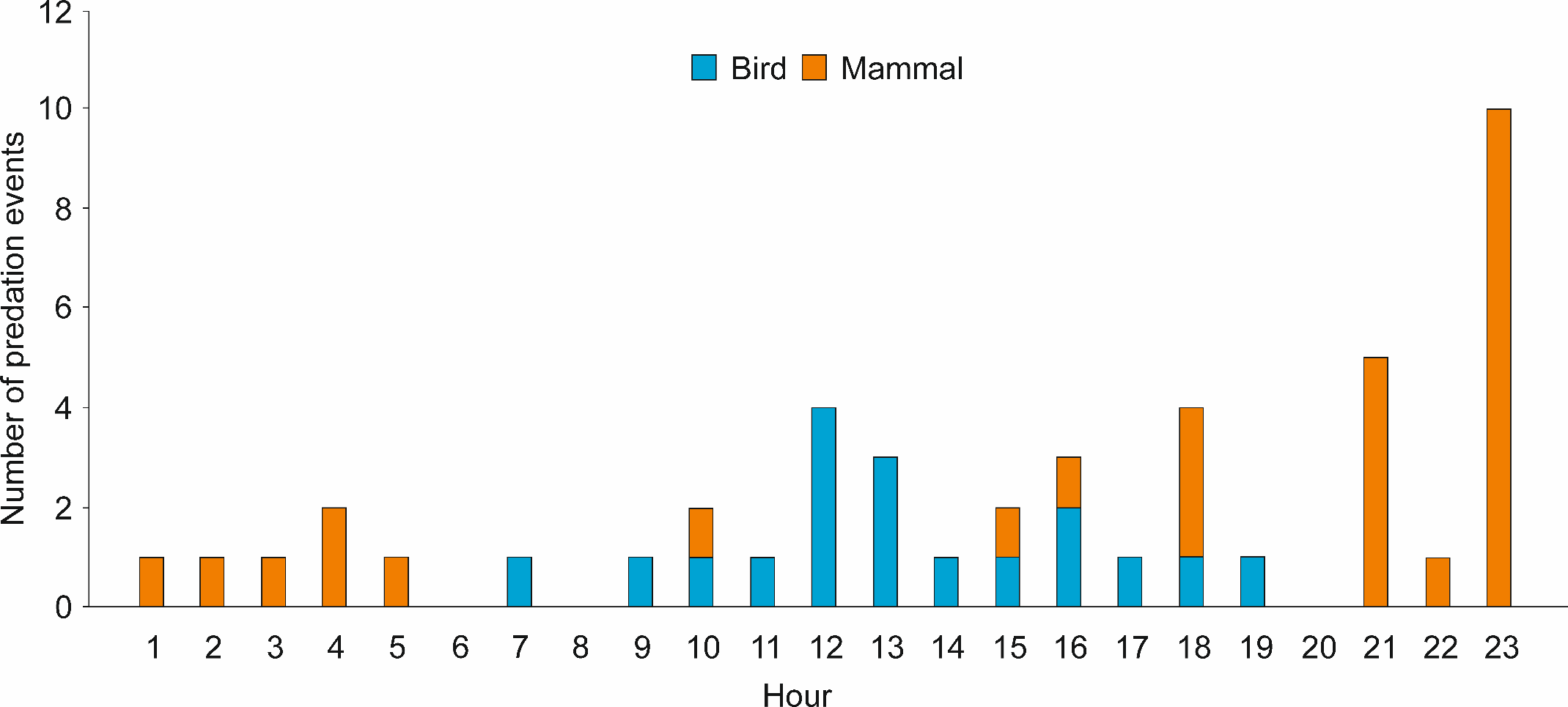

Supplement: Supplemental Information 2 [file peerj-12-17235-s002.docx]
